# COVID-19 associated bacterial infections in intensive care unit: a case control study

**DOI:** 10.1038/s41598-023-39632-2

**Published:** 2023-08-16

**Authors:** Bahar Kaçmaz, Şiran Keske, Uluman Sişman, Sena Tuğana Ateş, Mustafa Güldan, Yeşim Beşli, Erhan Palaoğlu, Nahit Çakar, Önder Ergönül

**Affiliations:** 1 Department of Infectious Diseases and Clinical Microbiology, American Hospital, Istanbul, Turkey; 2 Department of Infectious Diseases and Clinical Microbiology, Koc University School of Medicine, Istanbul, Turkey; 3 Koc University İşbank Center for Infectious Diseases, Istanbul, Turkey; 4 Koc University School of Medicine, Istanbul, Turkey; 5 Department of Clinical Laboratory, American Hospital, Istanbul, Turkey; 6 Intensive Care Unit, American Hospital, Istanbul, Turkey

**Keywords:** Microbiology, Diseases

## Abstract

We described the secondary bacterial infections (SBI) among COVID-19 patients in comparison with non-COVID-19 patients. We performed a retrospective case–control study between January 01, 2020 and April 01, 2022. Including the adult patients, who stayed ≥ 72 h in intensive care unit (ICU). In total 405 patients were included, 135 had (33.3%) COVID-19, with similar age and gender. The length of stay in ICU was not different (11.4 vs 8.2, *p* = 0.109), however mean intubation days were higher among COVID-19 cases (6.5 vs 3.8, *p = *0.005), SBI were more common among COVID-19 cases (34% vs 10.7%, *p* < 0.001). Among the patients with pneumonia, the rate of gram-positive bacteria was higher in COVID-19 group than the control group (39% vs 5%, *p = *0.006). The predictors for SBI were having COVID-19 (OR: 2.3, Cl 1.25–4.32, *p = *0.008), days of intubation (OR: 1.05, Cl 1.01–1.10, *p = *0.004), and being male (OR: 2, Cl 1.12–3.58, *p = *0.018). The predictors of mortality were COVID-19 (OR: 2.38, Cl 1.28–4.42, *p = *0.006), days of intubation (OR: 1.06, Cl 1.03–1.09, *p* < 0.001), active hematologic malignancy (OR: 3.1, Cl: 1.33–7.28, *p = *0.09), active solid tumors (OR: 2.44, Cl 1.21–4.91, *p = *0.012), and coronary artery diseases (OR: 1.8, Cl 1.01–3.52, *p = *0.045). The most common SBI in COVID-19 patients were methicillin-sensitive *Staphylococcus aureus.* No carbapenem-resistant Enterobacterales related infections were detected in COVID-19 patients.

## Introduction

The Coronavirus Disease 2019 (COVID-19) has a wide spectrum of clinical presentations from mild infection to respiratory failure that requires admission to the intensive care unit (ICU)^1^. Although the mortality rate has been decreased based on global vaccination, newly emerged variants with milder disease, and improved therapeutic options^2^, ICU admission might still be required among high-risk patients^3^. The mortality rate of COVID-19 in ICU at the initial months of pandemic ranged from 30 to 50%^4^ depending on the level of the ICU, mechanical ventilation, and study populations^5,6^. The main risk factors for mortality among the patients with COVID-19 in ICU are older age, male gender, hematologic and oncologic malignancy, interstitial lung disease, chronic kidney disease, diabetes mellitus, obesity, hypertension, and secondary bacterial infections^7,8^.

Secondary bacterial infections are one of the leading causes of mortality in ICUs. Secondary bacterial infections might occur depending on the status and duration of intubation, duration of ICU stay and catheter insertion^9^. Additionally, the use of corticosteroids and anti-cytokine medicines affect immunity and facilitate secondary infections^10^. In respiratory viral infections including influenza, the impact of secondary bacterial infections is a well-known issue^11^, however, its role in COVID-19 is complicated and still has uncertainties. In a meta-analysis, the rate of secondary bacterial infection among ICU patients was 8.1% (ranging from 0 to 25%)^12^. In this study, we aimed to investigate the etiology of secondary bacterial infections in COVID-19 and to describe the attributable effect of secondary bacterial infections on mortality in the ICU, in comparison with non-COVID-19 patients.

## Materials and methods

### Study design and population

This is a retrospective observational case–control study. From January, 1 2020 to April, 1 2022, we included patients older than18 years of age, and who stayed ≥ 72 h in intensive care unit (ICU). The cases were diagnosed, and laboratory confirmed by SARS-CoV-2 RNA RT-PCR, and the patients in the control group were COVID-19 negative (non-COVID-19). Infection diagnosed during admission to ICU was accepted as co-infection, and the infection detected after the admission to ICU was considered as secondary infection. All the bacteria causing bloodstream infections (BSI), respiratory tract infections and urinary tract infections (UTI) detected by cultures or molecular tests in the first episode were included. The outcomes of the study were in-hospital fatality and development of secondary infections. The patients who were transferred from other hospitals and the patients with any infections other than bloodstream, respiratory and urinary sources were excluded.

### Microbiological methods

In our hospital, automated blood culture system (BacT/ALERT 3D system, bioMérieux, France) is used. For identification of the bacteria, conventional laboratory methods and Matrix-Assisted Laser Desorption Ionization Time-of-Flight (VITEK^®^ MS system, bioMérieux, France) were used. Antimicrobial susceptibility test results were interpreted based on the European Committee on Antimicrobial Susceptibility Testing (EUCAST) recommendations. Besides, for defined cases that clinically requires an urgent result, lower respiratory tract samples (sputum, endotracheal aspirate specimens), were enrolled with BioFire^®^ FilmArray^®^ Pneumonia plus (bioMérieux, France).

As for SARS-CoV-2 RNA RT-PCR*,* nucleic acid extraction was performed using EZ1 Virus Mini Kit v2 (Qiagen, Hilden, Germany) on the EasyOne DNA isolation system (Qiagen, Hilden, Germany). Bio-Speedy SARS-CoV-2 þ Omicron RT-qPCR (Bioeksen R&D Technologies, Istanbul, Turkey) was used for the detection of SARS-CoV-2 ORF1ab&N genes; PCR amplification and analysis were performed on a Bio-Rad CFX96 Real-time System (BioRad, Hercules, CA, USA). The samples with the cycle threshold (Ct) value < 33 were considered positive for SARS-CoV-2 RNA.

### Definitions

*Ventilator-associated pneumoniae (VAP):* VAP refers to pneumonia that arises at least 48 h after mechanical ventilation. The possible VAP definition is met with the presence of purulent respiratory secretions which was defined as secretions from the lungs, bronchi, or trachea that contain ≥ 25 neutrophils and ≤ 10 squamous epithelial cells per low-power field (× 100) from ≥ 1 specimen collection^13^ or a positive lower respiratory tract culture (showing any growth); the probable VAP definition requires a positive culture of endotracheal aspirate, ≥ 10^5^ colony-forming units (CFU/mL) or equivalent semiquantitative result and positive culture of bronchoalveolar lavage, ≥ 10^4^ CFU/mL in addition to purulent secretions (from ≥ 1 specimen collection)^13^.

C*atheter related blood-stream infection (CRBSI)*: At least 48 h after the catheter insertion, CRBSI is based on blood cultures (obtained prior to initiation of antibiotic therapy) of the same organism from at least two blood samples, one from the catheter line and one from the peripheral vein^14^. Secondary CRBSI were excluded.

*Catheter-related urinary tract infection (CRUTI*): CRUTI refers to UTIs occurring in a person whose urinary tract is currently catheterized or had a catheter for at least 48 h^15^. If an indwelling urinary catheter was in place for more than two consecutive days in an inpatient location and then removed, the date of event for the UTI must be the day of device discontinuation or the next day for the UTI to be catheter-associated^15^. Patient should have at least one of the urinary symptoms or fever (> 38 °C) and has a urine culture with no more than two species of organisms identified, at least one of which is a bacterium of ≥ 10^5^ CFU/ml^15^.

Demographic characteristics, medical comorbidities, APACHE II scores, source of infections, culture results and antimicrobial sensitivity tests, laboratory tests (maximum C-reactive protein and procalsitonin in ICU stay), antibiotics and steroid use and clinical results were obtained for case and control group. Furthermore, for COVID-19 cases remdesivir, favipiravir, hyroroxychloroquine (HQ), lopinavir-ritonavir, oseltamivir, azithromycin use and immunomodulator treatments (steroid, tocilizumab and anakinra) were evaluated.

The study design and all experimental protocols were approved by Koç University Ethics Committee (Approval Number: 2022.352.IRB1.133) and due to retrospective study, informed consent was waived by the Koç University Ethics Committee, Istanbul, Turkey.

### Statistical analysis

For the comparison of categorical variable Chi square test, and for the continuous variables t-test were used. The multivariate analyses for both secondary infections and fatality were performed. The statistical significance was set as *p* < 0.05. STATA v. 16 was used.

### Ethics approval

The study design and all procedures performed in studies involving human participants were in accordance with the ethical standards of the Koç University Ethics Committee (Approval Number: 2022.352.IRB1.133).

## Results

**Figure 1:**
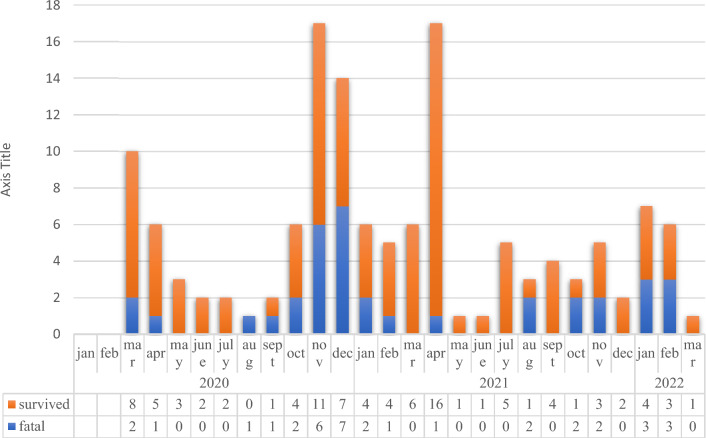
Number of the survived and fatal COVID-19 cases in intensive care unit.

We included 135 COVID-19 cases and 270 non-COVID-19 patients for control group (Table 1). The fatal and survived COVID-19 cases from the beginning of the pandemic (March of 2020) until the March of 2022 were presented in Fig. 1.

**Table 1:** Comparison of COVID-19 cases and the control group.

	COVID-19 n = 135 (%33.3)	Non-COVID-19 n = 270 (%66.7)	*p*
Mean age	71.8 (sd:15.2)	74.7 (sd:15.2)	0.077
Being male, gender	81 (60)	135 (50)	0.067
Comorbidities			
Hypertension	81 (60)	145 (54.1)	0.260
Coronary artery disease	38 (28.1)	59 (22)	0.174
Diabetes mellitus	41 (30.3)	82 (30.6)	0.963
Chronic obstructive pulmonary disease	7 (5.1)	39 (14.5)	0.005
Chronic renal disease	14 (10.3)	35 (13)	0.436
Demantia	17 (12.5)	40 (14.9)	0.526
Active solid malignancy	10 (7.4)	66 (24.6)	< 0.001
Active hematologic malignancy	13 (9.6)	20 (7.4)	0.454
Autoimmune diseases	4 (2.9)	13 (4.8)	0.370
Body mass index > 30	39 (29)	54 (20)	0.049
APACHE II scor (mean)	19.3 (sd:8.4)	22.8 (sd:8)	< 0.001
Length of stay, intensive care unit (mean)	11.4 (sd:30)	8.2 (sd:8.4)	0.109
Mean C-reactive protein, maximum	168 (sd:116)	189 (sd:127)	0.125
C-reactive protein ≥ 300 mg/L	17 (13)	55 (21)	0.050
Mean Procalcitonin, maximum	7.3 (sd:29)	13.6 (sd:36)	0.086
Procalcitonin ≥ 5 ng/mL	23 (17)	99 (37)	< 0.001
Days of intubation (mean)	6.5 (sd:9.7)	3.8 (sd:8.8)	0.005
Co-infections	67 (49.6)	151 (56.3)	0.202
Secondary infections	46 (34)	29 (10.7)	< 0.001
Pneumonia	31 (22.9)	16 (5.9)	< 0.001
Blood stream infection	19 (14)	12 (4.4)	0.001
Urinary tract infection	9 (6.7)	6 (2.2)	0.027
In-hospital mortality	36 (27%)	38 (14%)	0.006

The comorbidities were similar between two groups, but active solid tumor and chronic obstructive pulmonary disease (COPD) were significantly higher in control group (*p* < 0.001 and *p = *0.005). The mean APACHE II scor in ICU was higher among non-COVID-19 patients (*p* < 0.001). Although patients among body mass index > 30 was significantly higher in COVID-19 group (*p = *0.049), the frequencies of hypertension (60%, 54.1%) and diabetes mellitus (30.3%, 30.6%) were similar. The maximum levels of C-reactive protein (CRP) and procalcitonin among COVID-19 cases and non-COVID-19 cases were not significantly different (*p = *0.125 and *p = *0.086), but the levels of CRP ≥ 300 mg/L and procalcitonin ≥ 5 ng/mL are significantly higher in control group (*p = *0.05 and *p* < 0.001). The mean intubation days (*p = *0.005) was significantly higher in COVID-19 patients. The secondary infections were more commonly detected in COVID-19 cases (34%) than the control group (10.7%, *p* < 0.001). In both groups, the order of the most common secondary infections are pneumonia, blood stream infection and urinary tract infection. The comparison of COVID-19 cases and the control group are in Table 1.

Ventilator associated infections among pneumonia cases were 50% in COVID-19 cases and 56% in non-COVID-19 cases (*p = *0.284). Catheter related infections among bacteremia cases were detected in 30% of COVID-19 cases 40% of non-COVID-19 cases (*p = *0.567). In COVID-19 patients all the urinary tract infections were catheter related, whereas in non- COVID-19 infections 67% were catheter related.

Distribution of the agents isolated from the infections were presented in Table 2. Among the BSI infections, gram-positive bacteria, especially *Staphylococcus* spp. were the most commonly isolated bacteria in COVID-19 patients and the control group (*p = *0.362). Among the patients with pneumonia, the rate of gram-positive bacteria was higher in COVID-19 group than the control group (39% vs 5%, *p = *0.006, Table 2). Neither *Acinetobacter* spp. nor carbapenem-resistant Enterobacterales related infections were detected in COVID-19 patients (Table 2). The carbapenem resistance among *Acinetobacter baumannii* (n = 2) was 100%, among *Klebsiella* spp. (n = 7) 0%, and among *Pseudomonas aeruginosa* (n = 8) 50%. The rate of methicillin resistance among *Staphylococcus* spp. (n = 23) was 86% and 8.7% were methicillin- resistant *Staphylococcus aureus.*

**Table 2:** Distribution of bacterial agents in blood stream infection, pneumonia and urinary tract infection.

	COVID-19 n = 71 agents in 135 patients			Non-COVID-19 n = 43 agents in 270 patients		
	Blood stream infection n = 21 (%)	Pneumonia n = 41 (%)	Urinary tract infection n = 9 (%)	Blood stream infection n = 13 (%)	Pneumonia n = 19 (5)	Urinary tract infection n = 6 (%)
**Gram Positive Bacteria**	**16 (76)**	**16 (39)**	**3 (33)**	**8 (62)**	**1 (5)**	**1 (17)**
*Staphylococcus* spp.	9					
* Staphylococcus aureus*	3	13		1	1	
CN s*taphylococci*	6			6		
*Enterococcus* spp.	6			1		
* Enterococcus faecalis*	5		2	1		1
* Enterococcus faecium*	1		1	–		–
*Streptococcus pneumoniae*	–	2		–	–	
*Corynebacterium striatum*	1	1		–		
**Gram Negative Bacteria**	**5 (24)**	**25 (61)**	**6 (67**)	**5 (38)**	**18 (95)**	**5 (83)**
*Klebsiella* spp.						
* Klebsiella pneumoniae*	1	5	2	1	5	1
* Klebsiella oxytoca*	1			–	–	
*Acinetobacter baumannii*	–	–	–	2*	2	1
*Escherichia coli*	1	7	4	–	3	3
*Pseudomonas aeruginosa*	1	5		–	4	
*Serratia marcescens*	–	1		1	–	
*Morganella morganii*	1	1		–	–	
*Enterobacter* spp.						
* Enterobacter cloacae*	–	–		1	1	
* Enterobacter aerogenes*	–	2		–	–	
*Stenotrophomonas maltophilia*	–	4		–	3	

CN, coagulase-negative;*Blood stream infections are related to pneumonia.

Significant values are in bold.

Among COVID-19 patients, 6 patients out of 135 COVID-19 cases (4.4%) received remdesivir, 57 out of 135 (42%) favipiravir, 15 (11%) hydroxychloroquine, 9 (7%) lopinavir-ritonavir, azithromycin 10 (7.4%), 8 (6%) oseltamivir, 84 (62.2%) tocilizumab, 7 (5.1%) anakinra and 117 (%86.6) steroid. In control group 50.7% used steroids.

Overall 74 patients (18.1%) died in ICU. The fatality rate among COVID-19 cases was 27%, whereas in control group it was 14% (*p = *0.002, Table 1). In multivariate analysis, the parameters significantly associated with secondary bacterial infections were COVID-19 infection (OR: 2.3, CI 1.25–4.32, *p = *0.008), intubation days (OR: 1.05, CI 1.01–1.10, *p = *0.004), being male (OR: 2, CI 1.12–3.58, *p = *0.018, Table 3).In another multivariate analysis for the predictors of fatality, COVID-19 infection (OR: 2.38, CI 1.28–4.42, *p = *0.006), intubation days (OR: 1.06, CI 1.03–1.09, *p* < 0.001), active solid malignancy (OR: 2.44, CI 1.21–4.91, *p = *0.012), active hematologic malignancy (OR: 3.1, CI 1.33–7.28, *p = *0.009) and coronary heart disease (OR: 1.8, Cl 1.01–3.52, *p = *0.045, Table 3) were found to be significantly associated with fatality.

**Table 3:** Predictors of secondary infections and fatality in intensive care unit.

Predictors of secondary infections in ICU	Univariate analysis			Multivariate analysis		
	OR	CI	*P*	OR	CI	*P*
COVID-19	3.5	2.15–5.87	< 0.001	2.3	1.25–4.32	0.008
Intubation, days	1.1	1.05–1.11	< 0.001	1.05	1.01–1.10	0.004
Being male	1.9	1.14–3.14	0.013	2	1.12–3.58	0.018
Body mass index > 30	1.7	1.02–3.01	0.039	1.7	0.93–3.26	0.080
Length of stay, intensive care unit	1.13	1.10–1.17	< 0.001	1.02	0.98–1.05	0.180
APACHE II score, mean	0.98	0.95–1.01	0.199	0.98	0.94–1.01	0.301
Age > 65	0.89	0.50–1.58	0.706	1.23	0.61–2.46	0.550
Steroid use	2.3	1.32–4.04	0.003	1.4	0.72–2.73	0.310
Active solid malignancy	0.7	0.40–1.48	0.437	1.07	0.50–2.29	0.850
Active hematologic malignancy	1.5	0.68–3.42	0.305	1.3	0.53–3.57	0.498
Predictors of fatality in ICU						
COVID-19	2.2	1.31–3.67	0.003	2.38	1.28–4.42	0.006
Secondary infection	2.43	1.39–4.26	0.002	1.41	0.71–2.80	0.322
Being male	1.25	0.75–2.08	0.390	1.10	0.62–1.97	0.727
Intubation, days	1.07	1.04–1.06	< 0.001	1.06	1.03–1.09	< 0.001
APACHE II score, mean	1.02	0.99–1.05	0.066	1.03	0.99–1.07	0.052
Active solid malignancy	1.64	0.90–2.98	0.099	2.44	1.21–4.91	0.012
Active hematologic malignancy	2.83	1.32–6.06	0.007	3.1	1.33–7.28	0.009
Coronary heart disease	1.9	1.14–3.39	0.015	1.8	1.01–3.52	0.045
Body mass index > 30	0.59	0.30–1.15	0.124	0.5	0.23–1.07	0.075
Age > 65	1.20	0.64–2.23	0.562	0.82	0.39–1.73	0.616

ICU, intensive care unit.

## Discussion

The overall secondary bacterial infection rate in our ICU was 20% during two years of the pandemic period. The secondary bacterial infections were more common among COVID-19 patients (34%) than the control group (10.7%) (OR: 3.64, CI 2.14–6.21, *p* < 0.001) and pneumonia was the most common infection in both groups. Among COVID-19 cases intubation rate was 45% and VAP was 26%. In control group intubation rate was 57% and VAP was 5.8%. In one prospective study including 4244 critical COVID-19 patients the rate of intubation was 80% and VAP was %58^16^. In another study among intubated COVID-19 patients the secondary infection rate was reported as 43% and VAP as 78%^17^. Another study reported the secondary infection rate as 30% and VAP rate as 57.9% among COVID-19 patients in ICU^18^. The VAP rate among COVID-19 patients was higher than non-COVID-19 cases, however the intubation and the VAP rate in our study was much lower than previously reported studies. In two recent meta-analyses for the effectivity of tocilizumab, the authors reported the reduced use of mechanical ventilation and mortality^19,20^. The low rate of intubation and VAP in our study could be related with early administration of tocilizumab among severe COVID-19 cases. In our study the BSI rate among COVID-19 cases was 14%, and 30% was catheter-related. In one study reported BSI rate as 56%, and 25% of them were catheter-related^21^, and another study among COVID-19 cases the rate of BSI was 10%^17^. Our overall low secondary infection rate could be associated with effective infection control measures including high nurse/patient ratio, having isolated rooms for one patient, high rate of hand hygiene among the healthcare workers (85–90%), having bundles for infection prevention, and efficient antimicrobial stewardship program including rapid molecular diagnostic methods.

Overall fatality rate in our ICU was 18.1% and it was two times higher among COVID-19 patients than the control group (27% vs 14%, *p = *0.006, Table 1). The fatality rate among COVID-19 cases was 31.7% in 2020, 17.5% in 2021, 42.8% in the first 3 months of 2022 and the fatality rate was 47.5% for COVID-19 cases and 21% for control groups among intubated patients. Other studies reported more than 30% of fatality among COVID-19 patients in ICU^16,18,21^. Secondary bacterial infections were more common among COVID-19 infections, however in our study, the impact of secondary bacterial infections on fatality was not found to be statistically significant (Table 3). In our study, other than secondary bacterial infections such as COVID-19 infection itself, being intubated, hematologic malignancies, solid malignancies and coronary heart disease were found to be significantly associated with fatality. In a study from Turkey, secondary infection rate was reported as 29.7% among COVID-19 cases and the mortality as 52.5% in ICU^22^. Gram negative pathogens were the most common cause (72.5%) of secondary infections and carbapenem resistance rate was 62.1%^22^. In our hospital, *Acinetobacter baumannii* infections were eliminated^23^, although *A. baumannii* infections are common in the geographical region. In our study, only two patients in the control group had *A. baumannii* infections. Carbapenem resistant *Klebsiella* spp. infections are an important problem in Turkey^24^ however in our study, carbapenem resistance among *Klebsiella* spp. was not detected. Overall low rate of fatality among our patients could be associated with the relatively low rate of antibiotic resistance in our hospital.

In our hospital antiviral treatment (such as remdesivir), and immunomodulator treatments (corticosteroids, IL-1 and IL-6 inhibitors) are used by the clinical guidelines^25^. Based on our local algorithm, we used tocilizumab in severe COVID-19 patients from the beginning of the pandemic which improved survival and other clinical outcomes^26,27^, and its effectiveness was shown by the RECOVERY study team^28^. Favipiravir, hydroxychloroquine, lopinavir-ritonavir, and azithromycin were used only in the first few months of the pandemic. We did not use pulse steroids in any conditions among COVID-19 cases and our study showed that steroid use wasn’t predictor of secondary infection (OR: 1.4, Cl 0.72–2.73, *p = *0.310). In a systematic review it was reported that immune-based therapy for COVID-19 did not increase but decrease the risk of infections^29^.

The strong part of our study design was inclusion of full periods from January 2020 and April 2022, therefore having consequent patients with sufficient sample size. However, it was a single-center retrospective study as the most important limitation. Another limitation was inclusion of the first episodes of the secondary infections and we included only the most common categories of infections, such as BSI, pneumonia, and urinary system infections.

## Conclusion

In our study the secondary infections were found to be more common among COVID-19 patients, but had no effect on mortality. The most common bacteria were methicillin-sensitive *S. aureus.* Neither *Acinetobacter* spp. nor carbapenem-resistant Enterobacterales-related infections were detected in COVID-19 patients. The lack of association between secondary infections and fatality could be explained by our lower frequency of highly resistant gram-negative bacteria. This could be explained by the implementation of effective infection control measures including high nurse/patient ratio, having isolated rooms for one patient, high rate of hand hygiene among the healthcare workers, having bundles for infection prevention and efficient antimicrobial stewardship program including rapid molecular diagnostic method.

## Data Availability

The datasets generated during the current study are not publicly available due to the hospital’s personal data protection policy but are available from the corresponding author on reasonable request.
